# Concurrent naxitamab and lorlatinib in a patient with refractory high-risk neuroblastoma: a case report

**DOI:** 10.3389/fonc.2026.1790294

**Published:** 2026-03-23

**Authors:** Sultan Aydin, Ayse Oz

**Affiliations:** Pediatric Hematology and Oncology, Antalya Training and Research Hospital, Antalya, Türkiye

**Keywords:** lorlatinib, naxitamab, neuroblastoma, pediatric, refractory and/or relapsed high-risk neuroblastoma

## Abstract

Naxitamab is an anti-GD2 antibody currently approved in the United States as a treatment option for refractory and/or relapsed (R/R) high-risk (HR) neuroblastoma. With the identification of various mutations in tumor tissue by next-generation sequencing, patients with cancer increasingly have access to targeted therapies, including those directed at anaplastic lymphoma kinase (ALK) mutations. Here, we report a 12-year-old patient with refractory HR stage M neuroblastoma according to the International Neuroblastoma Risk Group staging system, with persistent bone and bone marrow involvement for 3 years, who was successfully treated with combined naxitamab and lorlatinib. After an ALK mutation was identified in the primary tumor tissue using the Pillar OncoReveal Multi-Cancer CNV RNA Fusion Panel, lorlatinib was initiated. Naxitamab therapy was subsequently added because of ongoing bone and bone marrow involvement. Our patient achieved complete remission with the combination of naxitamab and lorlatinib according to the International Neuroblastoma Response Criteria. Mild adverse events occurred during the combined immunotherapy, including transient hypotension managed with intravenous fluids and abdominal and leg pain that improved with analgesics. In conclusion, our case showed that combination therapy with naxitamab and lorlatinib may improve long-term outcomes and reduce chemotherapy-related toxicity.

## Introduction

Neuroblastoma is the most common extracranial solid tumor in childhood. It accounts for approximately 10% of all pediatric cancers and 15% of cancer-related deaths in children. Approximately 50% of neuroblastomas are classified as high-risk (HR) because of the presence of metastatic disease, most often involving the bone or bone marrow. Despite advances in treatment, outcomes remain poor, particularly for patients with refractory and/or relapsed (R/R) disease. Thus, additional therapeutic options are needed (1).

In recent years, immunotherapy has provided new treatment options for cancer. In pediatric solid tumors, however, its application remains limited compared with adults, largely because relatively few tumor-specific antigens have been identified (2). Neuroblastoma represents an exception. Over the past two decades, the discovery of monoclonal antibodies that target neuroblastoma-specific surface antigens known as gangliosides, and their subsequent clinical use, has significantly improved outcomes in HR neuroblastoma (3, 4).

Anti-disialoganglioside 2 (anti-GD2) antibodies may mediate lysis of neuroblastoma cells through interaction between C1q and the antibody. However, GD2-expressing afferent neurons may also undergo lysis (4). Another proposed mechanism is antibody-dependent phagocytosis. Monoclonal antibodies such as anti-GD2 can activate macrophages through various Fc receptors, thereby facilitating phagocytosis of neuroblastoma cells. The FcγRIIA (R/R) polymorphism in macrophages has been associated with improved progression-free survival in patients receiving anti-GD2 combined with granulocyte–macrophage colony-stimulating factor (GM-CSF) (3, 5). Naxitamab is a humanized (IgG1) anti-GD2 (hu3F8) monoclonal antibody that was developed for the treatment of neuroblastoma, osteosarcoma, and other GD2-positive malignancies. It was recently approved by the US Food and Drug Administration for use, in combination with GM-CSF, in pediatric patients aged 1 year and older and in adults with R/R HR neuroblastoma in the bone or bone marrow who achieved a partial response, minor response, or stable disease after prior therapy (6). Naxitamab has been rapidly incorporated into neuroblastoma treatment protocols because of its demonstrated ability to enhance treatment responses in clinical studies.

Mutations in the anaplastic lymphoma kinase (ALK) receptor tyrosine kinase are present at diagnosis in approximately 10% of cases, most commonly at positions F1174, F1245, or R1275 within the tyrosine kinase domain. Tyrosine kinase inhibitors (TKIs) are used as targeted therapy for oncogenic ALK mutations. Although some patients have achieved complete responses, the first- and second-generation ALK inhibitors crizotinib and ceritinib have often demonstrated limited clinical efficacy. By contrast, lorlatinib, a third-generation ALK TKI, is highly effective against a broad range of ALK mutant variants expressed in neuroblastoma (7, 8).

We herein report the effective combination of lorlatinib and naxitamab in a 12-year-old patient with ALK-mutated, refractory HR neuroblastoma involving refractory bone and bone marrow disease who was followed for 4 years.

## Case presentation

A 12-year-old girl with no known pre-existing conditions presented with a 1-year history of intermittent chest and back pain. She had no fever or respiratory distress. On initial examination, breath sounds were decreased in the left lung. The liver and spleen were palpable 2 cm below the costal margin. No lymphadenopathy was detected. Chest X-ray showed a mass occupying the left upper lung.

Laboratory results at presentation included a hemoglobin level of 12 g/dL, platelet count of 440,000/mm³, leukocyte count of 7,000/mm³, and neutrophil count of 2,100/mm³. Serum ferritin was elevated at 106 ng/mL (reference range, 10–30 ng/mL), lactate dehydrogenase was 471 U/L, uric acid was 3.3 mg/dL, and the erythrocyte sedimentation rate was 28 mm/h. Serum neuron-specific enolase (NSE) was markedly elevated at 283 µg/L (reference range, 1–15 µg/L).

**Figure 1:**
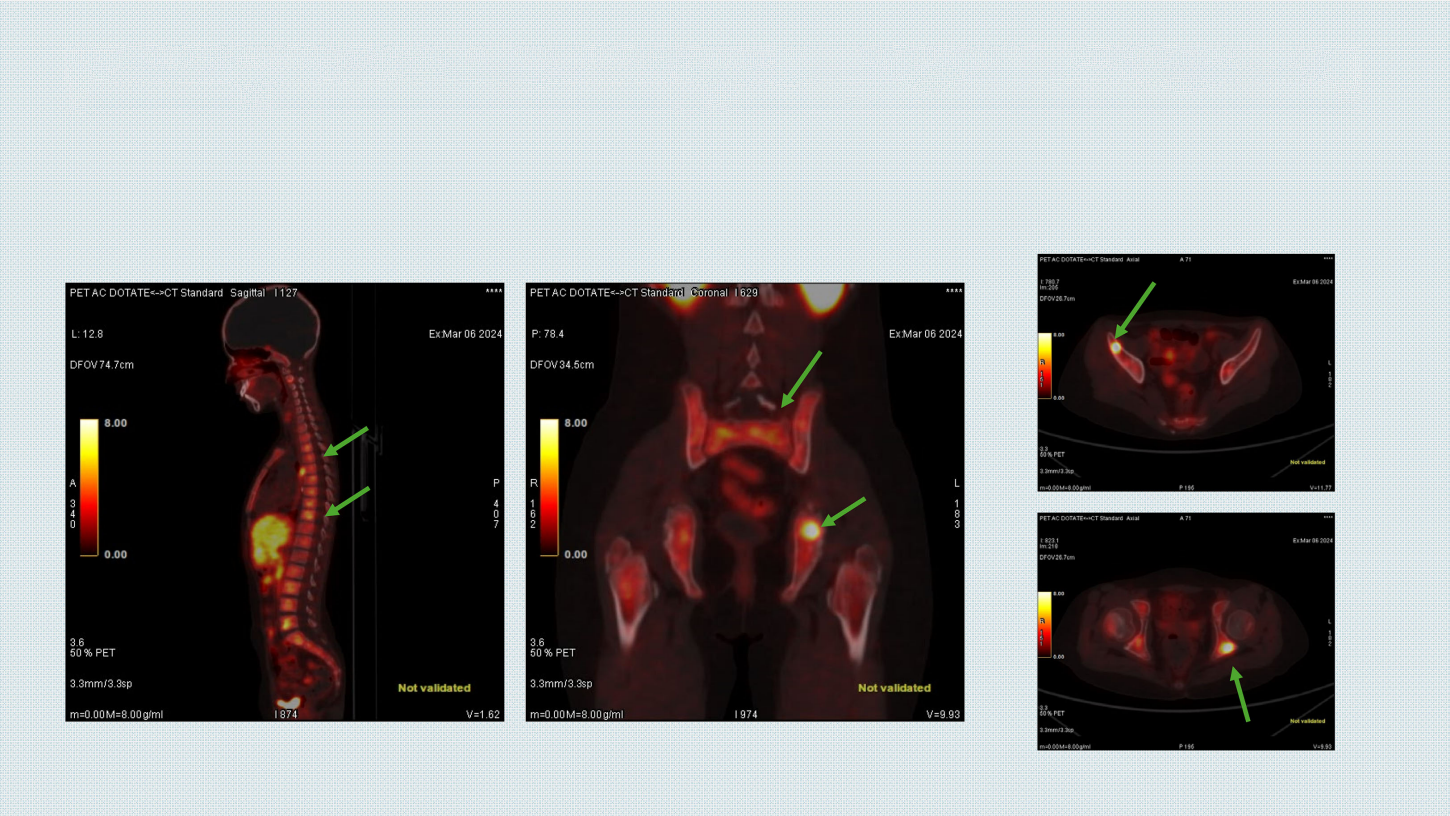
Bone involvement on DOTA-PET/CT image before combination treatment with lorlatinib and naxitamab.

Thoracic computed tomography (CT) revealed a mass measuring 76 × 74 × 82 mm in the posterior segment of the left upper lobe. Tumor biopsy was reported as neuroblastoma, and bone marrow biopsy confirmed neuroblastoma involvement. Tumor tissue was negative for N-myc amplification and 11q23 deletion, and the DNA index was near-diploid. Gallium-68 DOTA-positron emission tomography (PET)/CT identified bone involvement, with DOTA-positive uptake observed between the Th6–7 vertebrae, showing linearity with prevertebral localization (maximum standardized uptake value [SUV_max_]: 12.9), in the left retrocrural region (SUV_max_: 12.4), and in the left 11th intercostal space posteromedially (paravertebral localization, SUV_max_: 8.3) (**Figure 1**).

The patient was classified as being in the stage M high-risk group because of bone and bone marrow involvement according to the International Neuroblastoma Risk Group staging system, as well as age over 18 months. In accordance with the 2009 Turkish Pediatric Oncology Group neuroblastoma protocol, she received six cycles of conventional chemotherapy with sequential A9 (vincristine, dacarbazine, ifosfamide, doxorubicin) and A11 (cyclophosphamide, etoposide, cisplatin), which were completed without complications. The tumor was then laparoscopically resected after six cycles. Autologous stem cell transplantation was not performed because of bone marrow involvement.

After this treatment, Gallium-68 DOTA-PET/CT showed focal uptake in the C2 vertebra and extensive involvement of the thoracolumbar spine, L5 vertebra, left scapula, sacrum, bilateral pelvic bones, and proximal femora. A repeat bone marrow biopsy confirmed 10% neuroblastoma metastasis. Serum NSE decreased to 42.3 µg/L.

The persistent marrow involvement led to two cycles of ifosfamide, carboplatin, etoposide (ICE) as second-line therapy. At that time, NM_004304.5: c.3520T>C (p.Phe1174Leu) (resistant to crizotinib) (variant allele fraction: 73.91%) and NM_004304.5: c.3512T>C (p.Ile1171Thr) (variant allele fraction: 42.0%) ALK mutations were detected in the primary tumor using the Pillar OncoReveal Multi-Cancer CNV RNA Fusion Panel.

Because Gallium-68 DOTA-PET/CT showed ongoing refractory bone and bone marrow involvement despite NSE decreasing to 15 µg/L after ICE, lorlatinib (100 mg daily, days 1–21) was administered sequentially with the TEC regimen (topotecan 1.5 mg/m²/day, days 1–5; etoposide 80 mg/m²/day, days 4–6; and cyclophosphamide 200 mg/m²/day, days 1–5). After six TEC cycles, Gallium-68 DOTA-PET/CT demonstrated progression, leading to fourth-line treatment with the TVD protocol (topotecan, vincristine, doxorubicin) and lorlatinib (100 mg daily, days 1–21). Five cycles of TVD resulted in a serum NSE level of 20 µg/L.

Because of ongoing stage M, HR neuroblastoma with persistent bone and marrow disease, the patient received naxitamab, an anti-GD2 monoclonal antibody, administered as 3 mg/kg daily intravenous 1-hour infusions on days 1, 3, and 5, with premedications including gabapentin and sargramostim (Leukine^®^) starting 5 days before and continuing throughout treatment, along with supportive medications. During the initial cycles, she experienced acute adverse effects, including severe abdominal pain, anxiety, flushing, hypotension, bronchospasm, and pain, which were managed with supportive care. Most symptoms resolved within 24 hours post-infusion. Post-infusion hypotension was transient and corrected with hydration.

**Figure 2:**
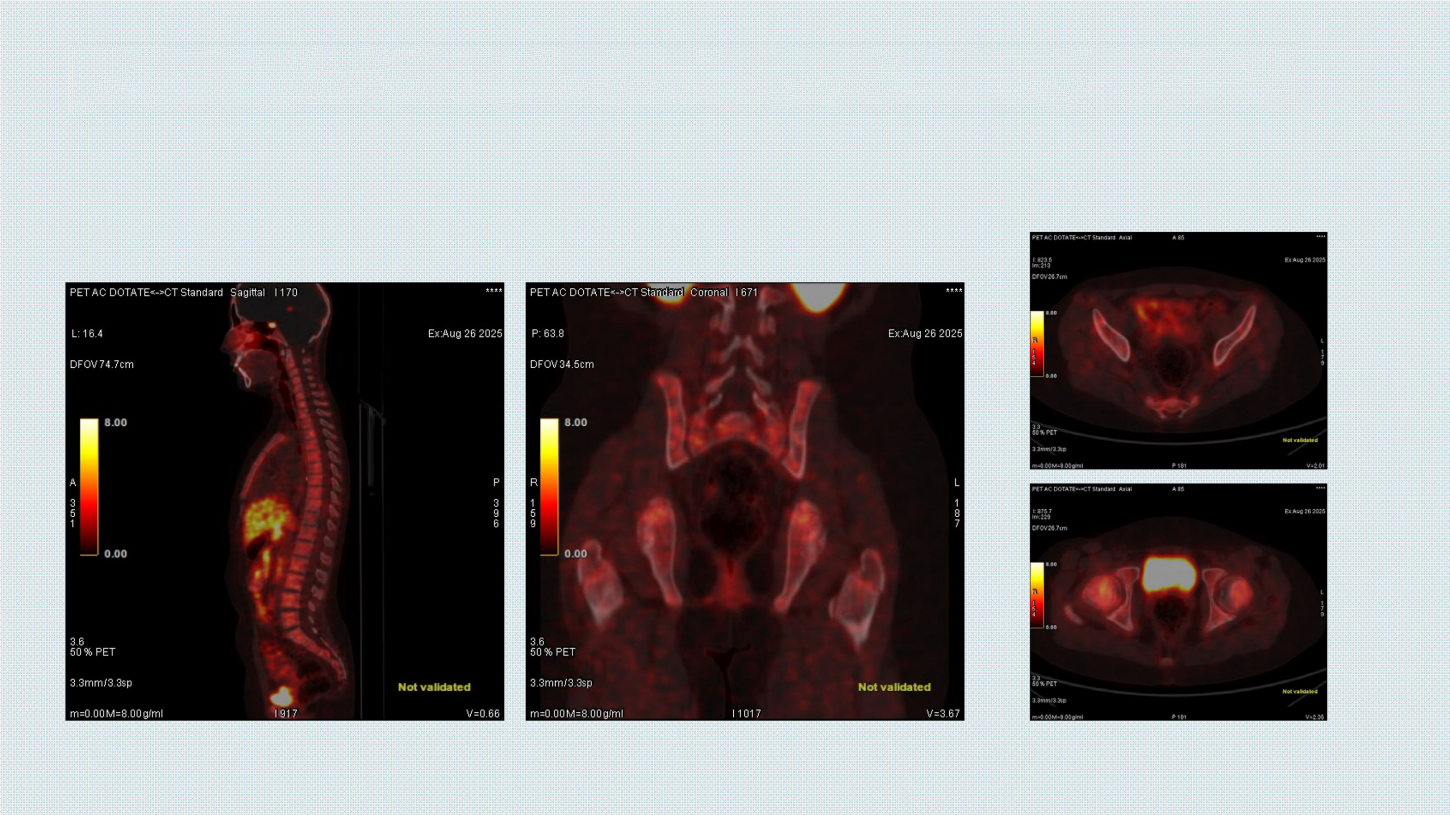
Improvement in bone involvement on DOTA-PET/CT image after combination treatment with lorlatinib and naxitamab.

**Figure 3:**
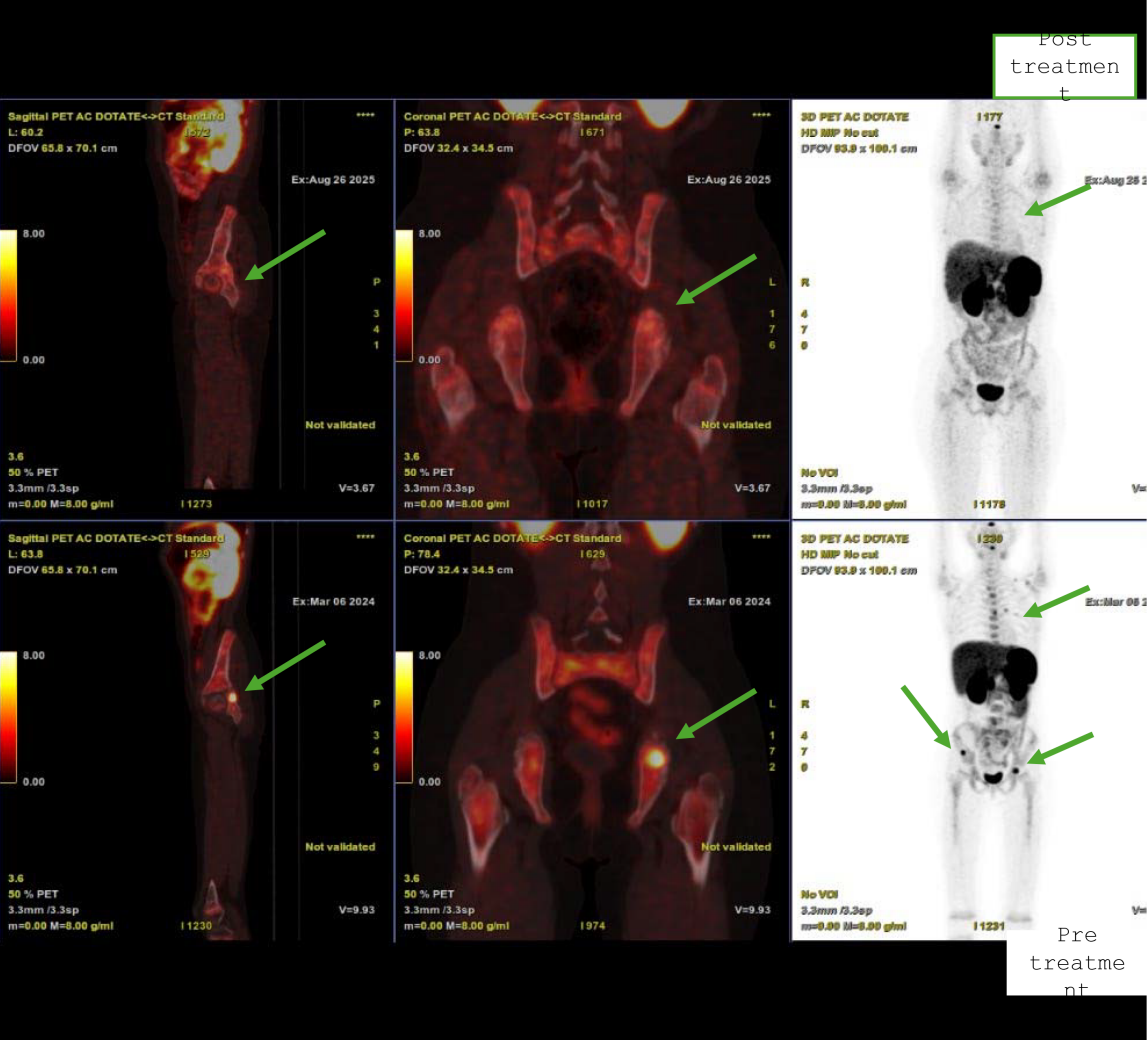
Regression of involvement in the iliac bone and thoracic vertebrae after combination treatment with lorlatinib and naxitamab.

Subsequent naxitamab cycles were associated with reduced pain, and all infusions were completed within 30 minutes without interruption. Gallium-68 DOTA-PET/CT scans after 10 cycles of combination lorlatinib and naxitamab showed low or negative DOTA uptake in the right anterior iliac bone (SUV_max_: 3.3), left posterior acetabulum (SUV_max_: 2.0), right pubic bone (SUV_max_: 2.4), and C2 vertebra (SUV_max_: 2.0), most prominently in the vertebral column, pelvic bones, and left scapula (**Figure 2**). Compared with the previous examination, decreased DOTA receptor activity was observed in the lesions, while DOTA receptor activity remained stable in other areas (**Figure 3**).

Bone marrow biopsy showed no marrow involvement, and the NSE level decreased to 7.32 µg/L. Ten cycles of combination naxitamab and lorlatinib treatment were successfully completed. At the time of this writing, the patient has been followed in complete remission according to the International Neuroblastoma Response Assessment Criteria for 1 year. Lorlatinib administration was interrupted for 1 week during each naxitamab treatment protocol and other chemotherapy protocols. Mild thrombocytopenia (platelet count of 100,000/mm³), a grade 1 toxicity according to the National Cancer Institute Common Terminology Criteria for Adverse Events (CTCAE v5.0), was chronically observed during the combination of naxitamab and lorlatinib.

## Discussion

Naxitamab is a humanized, cytolytic monoclonal antibody that targets the glycolipid GD2, an adhesion molecule that is widely expressed on the cell membranes of neuroectodermal-derived tumors, including neuroblastoma. It induces tumor cell death primarily through antibody-dependent cellular cytotoxicity and complement-dependent cytotoxicity. Naxitamab has been shown to be cytolytic against neuroblastoma cells in culture and to induce objective responses in 34–45% of children and adults with refractory advanced neuroblastoma involving the bone and bone marrow. In 2020, naxitamab received accelerated approval for the treatment of pediatric patients aged 1 year and older and adult patients with R/R HR neuroblastoma in the bone or bone marrow (9). Recent studies aimed at improving the low survival rates have supported the use of immunotherapy with anti-GD2 monoclonal antibodies such as dinutuximab and naxitamab in patients with HR neuroblastoma. However, optimization of treatment regimens remains necessary to improve outcomes further (10).

In a study of 82 patients older than 18 months with stage M disease, five cycles of GM-CSF at 250 μg/m²/day were administered for 5 days (days −4 to 0), followed by GM-CSF at 500 μg/m²/day for 5 days (days 1–5) in combination with naxitamab at 3 mg/kg/day on days 1, 3, and 5. N-myc amplification was identified in 21 patients (25.6%), and bone marrow involvement was detected in 12 patients (14.6%). According to that study, the 5-year event-free survival and overall survival rates in patients with HR neuroblastoma who achieved complete remission at the end of induction were 57.9% and 78.6%, respectively. These findings contributed significantly to the evolving treatment approach, implying that autologous stem cell transplantation and high-dose chemotherapy may not be necessary to achieve long-term survival in certain patients (11).

In a study evaluating the side effect profile of naxitamab, the most common adverse effects were hypotension (98%), pain (96%), urticaria (83%), pyrexia (79%), bronchospasm (67%), tachycardia (63%), cough (58%), vomiting (52%), and nausea (50%) (12). Bronchospasm, pain, and hypotension were more frequently observed during the initial treatment cycles, and both bronchospasm and hypotension were more severe during the first infusion of each cycle. Serious adverse effects occurred in 19 patients (39%), and naxitamab was permanently discontinued in 4 patients because of serious adverse events (anaphylaxis, respiratory depression, urticaria, and hypotension). In 77% of patients, infusion rate adjustments and infusion interruptions or discontinuations were required because of adverse effects. Side effects typically occurred within 24 hours after infusion completion, most often within 3 minutes of infusion initiation, and resolved shortly after the infusion ended in most patients (13). In Study 201, a multicenter phase 2 study, pain was observed in 96% of patients, with grade 3 pain reported in 65%. Hypotension occurred in 98% of patients, and 63% experienced grade 3–4 hypotension. Hypotension during naxitamab infusion was generally associated with tachycardia and could be accompanied by skin rash (11).

In our patient, the most common adverse effects were transient hypotension, widespread abdominal and leg pain, and rash. The symptoms resolved shortly after the infusion was stopped. Hypotension resolved with intravenous hydration using normal saline and did not require permanent interruption of the infusion. The longest-lasting adverse effect was pain, which completely resolved within 24 hours. There was no need to discontinue the infusion. Adverse effects were managed with supplemental oxygen, fluids, antihistamines, and analgesics administered during the infusion. As emphasized in previous studies, the adverse effects in our patient were reversible and manageable with supportive care. Considering that treatment-related adverse effects subsided immediately after discontinuation, it may be advisable, for patient comfort, to administer and complete the infusion as efficiently as possible under close monitoring, without unnecessarily prolonging the treatment duration. In our case, no abnormalities in liver function tests were observed during any treatment cycle.

The rationale for combining lorlatinib with naxitamab lies in their non-overlapping yet potentially complementary mechanisms of action. Neuroblastoma frequently harbors activating mutations in the ALK pathway, making ALK inhibition a rational targeted therapeutic strategy. Lorlatinib is a third-generation ALK TKI with high potency against resistant ALK mutations and excellent central nervous system penetration, and it has demonstrated clinical activity in relapsed or refractory ALK-mutated neuroblastoma (14). The combination of ALK inhibition and anti-GD2 immunotherapy is biologically plausible because of their complementary mechanisms. While lorlatinib directly inhibits oncogenic downstream pathways such as PI3K/AKT and MAPK, thereby reducing tumor cell proliferation and survival signaling, anti-GD2 therapy such as naxitamab promotes immune-mediated cytotoxicity independent of ALK signaling status. Preclinical evidence implies that targeted kinase inhibition may modulate the tumor microenvironment and potentially increase tumor susceptibility to immune-mediated killing. A recent study reported that 4 of 20 patients developed greater than grade 2 pulmonary toxicity when lorlatinib was used together with anti-GD2 therapy (15, 16). Published prospective data specifically evaluating this combination remain limited; therefore, expected toxicities are largely inferred from the known safety profiles of each agent. Future prospective studies are warranted to evaluate the safety, optimal sequencing, and potential synergistic efficacy of this combination formally.

Treatment with the combination of lorlatinib and naxitamab resulted in decreased bone and bone marrow involvement, although our patient was nonresponsive to multiple intensive chemotherapy regimens. As the first center in our country to administer naxitamab treatment, which offers relative convenience due to its manageable side effect profile, we present this case as an example of successful use in HR stage M neuroblastoma with refractory bone and bone marrow involvement, and to contribute to the limited data available in the literature. In our patient with HR neuroblastoma with refractory bone and bone marrow involvement, who had received fifth-line intensified chemotherapy, treatment with 10 cycles of naxitamab was highly satisfactory in terms of short-duration infusions, rapid resolution of adverse effects after each cycle, progressively milder adverse effects with subsequent cycles, ease of administration, and reduction in both the duration and number of hospitalizations.

## Conclusion

In pediatric patients, targeted therapies and immunotherapies associated with a mild side effect profile, low drug-related toxicity, fewer hospital admissions, shorter hospital stays, and the ability to maintain school and social activities—while providing effective treatment—are particularly important alternatives to intensified chemotherapy regimens, which may cause severe adverse effects such as febrile neutropenia.

## Data Availability

The original contributions presented in the study are included in the article/supplementary material. Further inquiries can be directed to the corresponding authors.
